# Factors associated with low adherence to oral 5-aminosalicylic acid in patients with ulcerative colitis

**DOI:** 10.1371/journal.pone.0214129

**Published:** 2019-03-22

**Authors:** Jin Lee, Sam Ryong Jee, Hyung Wook Kim, Dong Hoon Baek, Geun Am Song, Won Moon, Seun Ja Park, Hyun Jin Kim, Jong Hoon Lee, Jong Ha Park, Tae Oh Kim, for the Busan Ulsan Gyeongnam Intestinal Study Group Society (BIGS)

**Affiliations:** 1 Department of Gastroenterology, Haeundae Paik Hospital, Inje University College of Medicine, Busan, South Korea; 2 Department of Gastroenterology, Inje University Busan Paik Hospital, Inje University College of Medicine, Busan, South Korea; 3 Department of Gastroenterology, Pusan National University Yangsan Hospital, Yangsan, South Korea; 4 Department of Gastroenterology, Pusan National University School of Medicine, Busan, South Korea; 5 Department of Gastroenterology, Kosin University College of Medicine, Busan, South Korea; 6 Department of Gastroenterology, Gyeongsang National University Changwon Hospital, Changwon, South Korea; 7 Department of Gastroenterology, Dong-A University College of Medicine, Busan, South Korea; National Institutes of Health, UNITED STATES

## Abstract

**Background/Aims:**

It is well known that 5-aminosalicylic acid (5-ASA) is the standard first-line treatment for ulcerative colitis (UC). Medication adherence is an important factor in the treatment of UC. We aimed to identify predictors of low adherence to oral 5-ASA in Koreans with UC.

**Methods:**

Between July 2017 and January 2018, we performed a multicenter, cross-sectional study across 6 University Hospitals in Korea. Medication adherence was assessed using the modified Morisky Medication Adherence Scale (MMAS-8) questionnaire. Our study included 264 patients with UC. Patients were requested to complete the self-reported MMAS-8 questionnaire and a survey assessing sociodemographic data. Adherence was categorized as low (scores<6), medium (scores 6–7), and high (score 8).

**Results:**

The mean age of patients was 44±14 years, women comprised 43.6% of the study population and 49.8% of the studied population showed low adherence to oral 5-ASA. Age <40 years, alcohol consumption, and current smoking were significantly associated with low adherence to oral 5-ASA (age <40 years: odds ratio [OR] 1.76, 95% confidence interval [CI] 1.04–2.96, p = .034; alcohol consumption: OR 1.66, 95% CI 1.00–2.74, p = .049; current smoking: OR 4.06, 95% CI 1.08–15.18, p = .038). When data were classified based on gender, we observed that only in men, alcohol consumption and current smoking showed a significant association with low adherence to oral 5-ASA (alcohol consumption: OR 2.14, 95% CI 1.08–4.23, p = .029; current smoking: OR 5.07, 95% CI 1.32–19.41, p = .018). In women, only age <40 years was significantly associated with low adherence to oral 5-ASA (age <40 years: OR 3.71, 95% CI 1.59–8.66, p = .002).

**Conclusion:**

Approximately 50% of patients with UC showed low adherence to oral 5-ASA. Predictors of low adherence were age <40 years, alcohol consumption, and current smoking habits. In men, alcohol consumption and current smoking were significant predictors of low adherence, whereas in women only age <40 years was significantly associated with low adherence.

## Introduction

Ulcerative colitis (UC) is a chronic gastrointestinal disease that causes long-lasting colonic and rectal inflammation. The annual incidence rates of UC range between 15.7 and 24.3 cases per 100,000 person-years in North America and Europe, respectively [1,2]. The incidence and prevalence of UC has been rising recently in Asians [1,3,4]. The disease course is unpredictable and marked by alternating periods of remission and relapse, which may require hospitalization. The risk of developing colorectal cancer (CRC) is directly proportional to the duration of UC and is associated with an approximately 20% lifetime risk of CRC [5]. Conventionally, 5-aminosalicylic acid (5-ASA) is the standard first-line drug to induce and maintain remission in patients with UC.

Adherence to medication plays a significant role in the management of patients with chronic disease, and non-adherence lowers the effectiveness of treatment and increases medical costs [6]. Adherence to oral 5-ASA is an important issue in determining disease activity in UC. Thus, it is essential to understand the degree of therapeutic adherence and factors that affect adherence. Based on previous studies [7–12], adherence to 5-ASA is observed to be poor in real-world clinical practice, ranging from 40–60% in patients with inflammatory bowel disease (IBD). Several studies [7–14] have reported a number of risk factors associated with non-adherence in patients with UC. These factors include age, gender, newly diagnosed patient status, disease duration, copayment costs, presence of psychiatric disease such as depression, concomitant use of 5-ASA suppository or glucocorticoid, and dosing regimens of oral 5-ASA. Notably, factors related to non-adherence differ across studies; however, it should be noted that these studies have used different tools for assessing adherence levels.

The 8-item Morisky Medication Adherence Scale (MMAS-8) is a simple, self-reported questionnaire that has been validated in patients with chronic diseases [15–17]. Previous studies [11,12] have used the MMAS-8 survey to assess non-adherence to treatment in patients with IBD. However, these previous studies report that treatment modalities used in patients with UC have included not only oral medications but also non-oral preparations. A few questions included in the MMAS-8 survey are not ideally applicable in relation to the administration of non-oral, nondaily medications such as subcutaneous injections or intravenous infusions. Furthermore, the survey does not include separate questions for each specific therapy. Previous studies included both groups of patients—those diagnosed with UC and also CD. Moreover, data obtained from Asia regarding treatment adherence in patients with IBD have not been studied in detail. Therefore, using the MMAS-8 survey, we investigated the prevalence of low adherence to oral 5-ASA and aimed to identify predictors of low adherence in Koreans with UC.

## Methods

### Study subjects

Between July 2017 and January 2018 we performed a multicenter, cross-sectional study across 6 University Hospitals in Korea. Consecutive patients diagnosed with UC over at least 6 months were prospectively recruited from 6 University Hospitals. Inclusion criteria for the study were: (1) Patients aged >15 years, (2) those currently being prescribed oral 5-ASA medication for >6 months, (3) those with a colonoscopy- and biopsy-proven diagnosis of UC. Our study included 264 patients with UC. All enrolled patients were requested to complete the MMAS-8 questionnaire and a survey comprising sociodemographic data. Ethical approval for this study was obtained from the Institutional Review Board of Inje University Haeundae Paik Hospital (HPIRB 2017-06-005-001). All participants provided written informed consent.

### Questionnaire

All enrolled patients were required to complete an MMAS-8 self-report questionnaire when they visited the outpatient clinic. Medication adherence to oral 5-ASA was assessed using the MMAS-8 scale, which has been validated in patients with chronic diseases [15–17]. The self-report questionnaire is simple and can be completed easily in a clinical setting. It contains 8 questions with the first 7 having a binary answer (yes or no) and a 5-point Likert scale for the last question. A negative answer to a question received a score of 1 point. The score for the last question was graded between 0 and 1 on the 5-point scale (never = 1, once in a while = 0.75, sometimes = 0.5, usually = 0.25, and all the time = 0). The total score is calculated as the sum of all MMAS-8 questions and ranges from 0–8. Adherence was categorized as low (scores <6), medium (scores 6–7), and high (score = 8). For this study, we introduced the term UC in each question of the MMAS-8 questionnaire, and the question was translated into Korean using forward and backward translation, based on the recommendations of Wild et al [18].

### Clinical variables

Clinical variables included sociodemographic data, lifestyle factors, clinical information related to disease and medication. Data regarding sociodemographic characteristics and lifestyle were obtained using a self-administered survey, which patients completed along with the MMAS-8 questionnaire. Sociodemographic data included age, gender, financial and education level, and marital status. Lifestyle variables included smoking, drinking, and exercise habits. Individuals who smoked were classified as current, former, or never smokers. A current smoker was defined as an individual who had smoked in the month prior to enrollment in the study. Alcohol users were categorized as drinkers or non-drinkers. Current alcohol use was defined as alcohol consumption >30 g/week. Enrolled patients were instructed to report their mean alcohol intake and the frequency of alcohol use as: never, once a month, 2–3 days monthly, 2–3 days weekly, or >4 days weekly.

We reviewed medical records to obtain clinical information related to disease and medication usage. Clinical information included duration of UC, the number of oral 5-ASA medications administered per day, the types of 5-ASA, the time periods of administration of oral 5-ASA medication per day, the status of anti-tumor necrosis factor (TNF) injections or 5-ASA suppositories, and the number of previous UC flare-ups. The types of 5-ASA included mesalamine (brand name: Mezavant, Pentasa, Asacol) and balsalazide (brand name: Colazal). The time since the diagnosis of UC was classified into 3 categories: <1 year, between 1 and 5 years, and >5 years. The number of oral 5-ASA medications administered was classified as: <6 drugs or >6 drugs. The frequency of medication was classified as once or >than twice. The UC flare up was defined as the occurrence of symptoms after a period of remission.

### Statistical analysis

Data have been presented as frequencies and percentages for categorical variables and means±standard deviations (SD) for numerical variables. Differences in patient characteristics were compared across subgroups using the chi-square test for categorical variables and the independent t test or the Mann-Whitney U test for continuous variables. We used the Shapiro-Wilk test to check for normality of distribution. Univariate and multivariate analyses were performed using logistic regression to identify prognostic factors that are independently related to low adherence to oral 5-ASA. All statistical analyses were performed using the SPSS software version 24.0. A p value <0.05 was considered statistically significant.

## Results

Of the 264 patients who enrolled in the study, 259 patients completed the questionnaire (response rate: 98%). The mean age of patients was 44.1 years (SD = 14.0) and 93 (35.9%), were aged <40 years. Among these, 113 patients (43.6%) were women, 14 (5.4%) were current smokers, and 117 (45.2%) consumed alcohol. We observed that 15% of the patients diagnosed with UC received anti-TNF therapy, 27% received 5-ASA suppository, and 18% received azathioprine. At the time of initial diagnosis of UC, 90 patients (34.7%) had been diagnosed with proctitis, 111 (42.9%) with left-sided colitis, and 58 (22.4%) with extensive colitis.

We observed low adherence to oral 5-ASA in 129 patients (49.8%). The mean MMAS-8 score was 5.5 (range 0.5–8). Notably, 129 patients (49.8%) showed a score of <6 (low adherence), 95 (36.7%) showed a score of 6 to <8 (medium adherence), and 35 (13.5%) showed a score of 8 (high adherence). Based on the answers provided in the MMAS-8 survey, the most common reason for nonadherence was “Patients feel hassled about sticking to the treatment plan” (47.9%), and the second most common reason was “Patients sometimes forget to take their pills” (44.8%).

### Univariate analysis of risk factors for low adherence to 5-aminosalicylic acid

Table 1 shows the sociodemographic characteristics and lifestyle factors related to the adherence rate. Univariate analysis showed that age <40 years, current smoking, and alcohol consumption were significantly associated with low adherence to oral 5-ASA (p = 0.034, 0.027, and 0.029, respectively). However, the frequency of alcohol consumption was not significantly related to low adherence (p = 0.240). Table 2 shows the disease- and medication-related variables related to the adherence rate. Disease-related factors such as disease duration and extent at the time of diagnosis, the presence of comorbidities, and the number of previous UC flare-ups were not associated with low adherence. Medication-related factors such as the number of medications, time of administration of medications, and types of 5-ASA drugs were also not related to low adherence.

**Table 1:** Socio-demographic characteristics and life style regarding to adherence rate.

Variable	Overall	Low adherence	Medium/High adherence	P
**All patients**	259 (100.0)	129 (49.8)	130 (50.2)	
**Age**	44.17±14.31	41.98±13.80	46.35±14.52	.014^1^
< 40 years	93 (35.9)	55 (42.6)	39 (30.0)	.034^2^
≥ 40 years	166 (64.1)	74 (57.4)	91 (70.0)	
**Sex**				
Male	146 (56.4)	67 (51.9)	79 (60.8)	.152^2^
Female	113 (43.6)	62 (48.1)	51 (39.2)	
**Smoking**				
Current	14 (5.4)	11 (8.5)	3 (2.3)	.027^2^
Former, never	245 (94.6)	118 (91.5)	127 (97.7)	
**Drinking state**				
Yes	117 (45.2)	67 (51.9)	50 (38.5)	.029^2^
No	142 (54.8)	62 (48.1)	80 (61.5)	
**Marital status**				
Single and ever married	70 (27.0)	40 (31.0)	30 (23.1)	.151^2^
Married	189 (73.0)	89 (69.0)	100 (76.9)	
**Exercise state**				
< 3 times/week	164 (63.3)	85 (65.9)	79 (60.8)	.392^2^
≥ 3 times/week	95 (36.7)	44 (34.1)	51 (39.2)	
**Education level**				
≤ Elementary	14 (5.4)	6 (4.7)	8 (6.2)	.678^2^
Middle, High	109 (42.1)	52 (40.3)	57 (43.8)	
≥ College	136 (52.5)	71 (55.0)	65 (50.0)	
**Socioeconomic status**				
High	13 (5.0)	7 (5.4)	6 (4.6)	.955^2^
Medium	206 (79.5)	102 (79.1)	104 (80.0)	
Low	40 (15.4)	20 (15.5)	20 (15.4)	

Values are either mean±SD or frequency with percentage in parentheses.

^1^ P values were derived from independent t-test.

^2^ P values were derived from chi-square test.

Shapiro-Wilk’s test was employed for test of normality assumption.

Use of the MMAS is protected by US Copyright laws. Permission for use is required. A license agreement is available from Donald E. Morisky, MMAS Research LLC 14725 NE 20th St. Bellevue WA 98007 or from dmorisky@gmail.com.

**Table 2:** Disease and medication-related variables regarding to adherence rate.

Variable	Overall	Low adherence	Medium/High adherence	P
**Disease duration in years**	5.89±4.87	5.51±4.06	6.27±5.54	.743^1^
< 1 year	20 (7.7)	10 (7.8)	10 (7.7)	.955^2^
1–5 years	125 (48.3)	63 (48.8)	62 (47.7)	
≥ 5 years	114 (44.0)	56 (43.4)	58 (44.6)	
**Number of oral 5-ASA / day**	3.69±1.78	3.78±1.74	3.61±1.82	.546^1^
1–5	205 (79.2)	100 (77.5)	105 (80.8)	.520^2^
≥ 6	54 (20.8)	29 (22.5)	25 (19.2)	
**Times of oral 5-ASA**				
Once a day	124 (47.9)	63 (48.8)	61 (46.9)	.758^2^
Twice or 3 times a day	135 (52.1)	66 (51.2)	69 (53.1)	
**5-ASA suppository**				
Yes	71 (27.4)	36 (27.9)	35 (26.9)	.859^2^
No	188 (72.6)	93 (72.1)	95 (73.1)	
**Anti-TNF**				
Yes	40 (15.4)	19 (14.7)	21 (16.2)	.751^2^
No	219 (84.6)	110 (85.3)	109 (83.8)	
**Azathioprine use**				
Yes	49 (18.9)	22 (17.1)	27 (20.8)	.445^2^
No	210 (81.1)	107 (82.9)	103 (79.2)	
**Types of 5-ASA**				
Mezavant	62 (23.9)	24 (18.6)	38 (29.2)	.256^2^
Pentasa slow	97 (37.5)	51 (39.5)	46 (35.4)	
Asacol dr	76 (29.3)	41 (31.8)	35 (26.9)	
Colazal	24 (9.3)	13 (10.1)	11 (8.5)	
**Disease extent at diagnosis**				
Proctitis	90 (34.7)	47 (36.4)	43 (33.1)	.710^2^
Left side colitis	111 (42.9)	52 (40.3)	59 (45.4)	
Extensive colitis	58 (22.4)	30 (23.3)	28 (21.5)	
**Presence of comorbidities**				
Yes	69 (26.6)	29 (22.5)	40 (30.8)	.131^2^
No	190 (73.4)	100 (77.5)	90 (69.2)	
**Number of UC flare up in past year**	0.45±0.93	0.44±0.90	0.46±0.97	.910^1^
0	192 (74.1)	95 (73.6)	97 (74.6)	.923^2^
1–2	52 (20.1)	27 (20.9)	25 (19.2)	
≥ 3	15 (5.8)	7 (5.4)	8 (6.2)	

Values are either mean±SD or frequency with percentage in parentheses.

^1^ P values were derived from Mann-Whitney’s U test.

^2^ P values were derived from chi-square test.

Shapiro-Wilk’s test was employed for test of normality assumption.

### Multivariate analysis of risk factors associated with low adherence to 5-aminosalicylic acid

Age <40 years, alcohol consumption, and current smoking were significantly associated with low adherence to oral 5-ASA (age <40 years: odds ratio [OR] 1.76, 95% confidence interval [CI] 1.04–2.96, p = .034; alcohol consumption: OR 1.66, 95% CI 1.00–2.74, p = .049). Additionally, current smoking showed a highly significant association with low adherence to oral 5-ASA (current smoking: OR 4.06, 95% CI 1.08–15.18, p = .038). When data were classified in terms of gender, only in men, alcohol consumption and current smoking showed a highly significant association with low adherence to oral 5-ASA (alcohol consumption: OR 2.14, 95% CI 1.08–4.23, p = .029; current smoking: OR 5.07, 95% CI 1.32–19.41, p = .018). In women, only age <40 years was significantly associated with low adherence to oral 5-ASA (age <40 years: OR 3.71, 95% CI 1.59–8.66, p = .002). Table 3 shows the results of multivariate analysis for low adherence to oral 5-ASA.

**Table 3:** Risk factor analysis for low adherence to oral 5-ASA.

	All patients		Male		Female	
Variable	OR (95% CI)	P	OR (95% CI)	P	OR (95% CI)	P
**Young age < 40 years**	1.76 (1.04–2.96)	.034	1.04 (0.51–2.12)	.915	3.71 (1.59–8.66)	.002
**Alcohol consumption**	1.66 (1.00–2.74)	.049	2.14 (1.08–4.23)	.029	1.25 (0.54–2.90)	.598
**Current smoking**	4.06 (1.08–15.18)	.038	5.07 (1.32–19.41)	.018	-^1^	-

OR: Odds ratio, CI: Confidential interval.

The multivariate model was created using all significant variables in univariate model in Table 1 and Table 2 as covariates.

^1^ All female patients were non-smokers.

## Discussion

The present study showed that medication adherence to oral 5-ASA was approximately 50% in patients with UC enrolled in this study. Our results are in agreement with previous studies that have reported prevalence of nonadherence to medication ranging from 40–60% [7–10]. Several previous studies have reported risk factors associated with low adherence to treatment in patients with IBD [7–14]. In this study, we found that age <40 years, alcohol consumption, and current smoking were significant predictors of low adherence to oral 5-ASA. When data were classified in terms of gender, we observed that only in men, alcohol consumption and current smoking showed a highly significant association with low adherence to oral 5-ASA. In women, only age <40 years was significantly associated with low adherence to oral 5-ASA.

Previous studies have demonstrated that young age is a significant factor associated with low adherence [9,14,19], and this result is in agreement with the findings of our study. This observation may be explained by the fact that UC primarily affects younger individuals who are active professionally and in social life. However, when data were classified in terms of gender, we observed that only in women, age <40 years showed a significant association with low adherence to oral 5-ASA. No significant association was observed between younger age and low adherence in men because smoking and alcohol consumption were observed to be more significant factors than younger age in men based on statistical analysis.

This study showed that alcohol consumption and current smoking were significant risk factors associated with low adherence to oral 5-ASA. Previous studies [20–25] have reported conflicting findings regarding the association between alcohol consumption and the risk of UC. Patients with UC who consume alcohol may experience different outcomes. A few patients report relapse in the form of a severe acute attack, whereas others are observed to be at a higher risk of liver injury. Alcohol is known to interact with several drugs including 5-ASA. Both, smoking and alcohol consumption play a significant role in the management of UC. However, cigarette smoking, despite its well-established negative effects on overall health, may show a positive effect in patients with UC [26–29]. Researchers propose that the positive effects of smoking in patients with UC may be attributable to the nicotine content of cigarettes. However, studies have not definitively described the effect of nicotine on UC and a beneficial effect, if any, is yet to be conclusively established.

In real-world clinical practice, the current recommendation is that patients with UC should avoid alcohol and smoking. The physician-patient relationship is an important factor in achieving higher treatment-adherence rates. Patients who continue to smoke and drink ignoring their physician’s advice to reduce or avoid these habits are considered to show low adherence to treatment. Usually, the percentage of smokers and alcohol drinkers is higher in men than in women. In our study, all women were non-smokers, and 66% of those consuming alcohol were men. Thus, gender could be a confounder in performing statistical analysis. In view of this consideration, we analyzed the adherence rate after classifying our data in terms of gender. Based on this analysis, we observed that only in men, alcohol consumption and current smoking showed a significant association with low adherence to oral 5-ASA.

There are conflicting data regarding factors including gender, marital status, education level, and physical activity as predictors of non-adherence in patients with UC [7–14,30,31]. Although a few studies have demonstrated that the male gender was significantly associated with nonadherence [7,30], Mackner et al. [29] have reported greater levels of low/non-adherence in young women. A few other studies have not reported any relationship between gender and nonadherence [10,19]. Similarly, a few studies have shown an association between a higher education level and nonadherence [9,10,13,32]. In this study, we did not observe any relationship between the afore-mentioned variables and nonadherence.

There are also conflicting data regarding the role of disease- or medication-related factors as predictors of non-adherence. For example, multiple daily doses or a large number of drugs are well-known factors associated with low adherence to oral medication. Recent studies [33–35] further support the use of once-daily treatment to improve medical compliance. However, in our study, medication-related factors such as the number of medications, the time of administration of the medications, and the types of 5-ASA administered did not show a significant association with low adherence. Disease-related factors such as disease duration and its extent at the time of diagnosis, the presence of comorbidities, and the number of previous UC flare-ups did not show a significant association with low adherence.

Strengths of our study: Although several previous studies describe nonadherence, data regarding non-adherence to treatment of IBD in Asians has not been studied in detail. 1) To our knowledge, this is the first study to evaluate the prevalence and predictors of low adherence to oral 5-ASA in Koreans diagnosed with UC. 2) The relatively large sample size in this study serves as a strength. 3) Patients receiving oral 5-ASA treatment were included in this study because a few questions included in the MMAS-8 survey are not ideally applicable to the administration of non-oral, nondaily medications such as subcutaneous injections or intravenous infusions, and the survey does not include separate questions for each specific therapy.

Limitations of this study:1) We could not confirm the accuracy/validity of the relationship between self-reported compliance and actual compliance because the MMAS score was not compared to pharmacy prescription records or hospital prescription records that could have accurately identified medications. 2) Medication adherence assessed via questionnaires is likely to show higher levels than actual levels because of social desirability among patients. Nonetheless, it has been shown that such a questionnaire can reliably identify non-adherence [36]. 3) MMAS scores may be affected by a recall bias because questionnaires only rely on a patient’s memory. 4) All female patients in our study were non-smokers. It is thought that the smoking rate of Korean women is still low compared to Europe or the US, and it may be difficult to apply our findings to the general population in other countries.

## Conclusion

In this study, age <40 years, alcohol consumption, and current smoking were observed to be predictors of low adherence to medication. In men, alcohol consumption and current smoking were significant predictors of low adherence. In women, age <40 years was the only factor that showed a significant association with low adherence. Healthcare providers ought to focus on improved patient education and close monitoring of medication adherence in young patients and in those presenting with known alcohol consumption and current smoking habits.
